# Nanocrystals: A perspective on translational research and clinical studies

**DOI:** 10.1002/btm2.10122

**Published:** 2018-12-24

**Authors:** Maria Jarvis, Vinu Krishnan, Samir Mitragotri

**Affiliations:** ^1^ Dept. of Bioengineering Rice University Houston TX 77030; ^2^ John A. Paulson School of Engineering and Applied Sciences Wyss Institute, Harvard University Cambridge MA 02138

**Keywords:** clinical trials, nanocrystals, nanomedicine, nanoparticles, nanotherapeutics, translational medicine

## Abstract

Poorly soluble small molecules typically pose translational hurdles owing to their low solubility, low bioavailability, and formulation challenges. Nanocrystallization is a versatile method for salvaging poorly soluble drugs with the added benefit of a carrier‐free delivery system. In this review, we provide a comprehensive analysis of nanocrystals with emphasis on their clinical translation. Additionally, the review sheds light on clinically approved nanocrystal drug products as well as those in development.

## 1 INTRODUCTION

Over the years, nanoparticles (NPs) made of both organic and inorganic materials have been engineered to circumvent the biological barriers and deliver drugs for a variety of indications.^1^, ^2^ Water‐insoluble or hydrophobic drugs, pose a challenge in terms of achieving optimal bioavailability and thereby, adequate efficacy.^3^ As reported in 2015, 40% of drugs on the market and 90% of drugs within the discovery pipeline face solubility issues.^4^ Other statistics, cite 40% of all potential drug candidates were shelved as a result of intrinsic aqueous solubility issues.^5^ Thus, a number of hydrophobic drugs, which could potentially be useful for treatments are in need of clinically acceptable carriers.^6^

**Figure 1:**
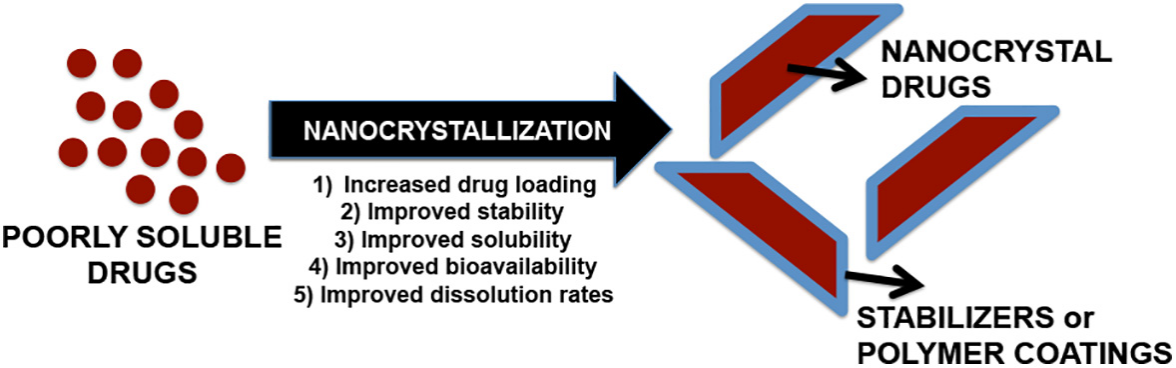
Nanocrystallization of poorly soluble drugs improves physicochemical stability and drug bioavailability

For the purpose of this review article, drug nanocrystals may be defined as pure solid particles with a mean diameter <1 μm and a crystalline character. The platform offers an exceptional opportunity to deliver hydrophobic drugs (Figure 1). Its uniqueness originates from the fact that nanocrystals are composed entirely of 100% drug or the payload thereby eliminating the ancillary role of a carrier.^7^ In addition, surfactants or stabilizers are commonly used to stabilize the crystalline dispersions in liquid media.

Nanocrystalline drug technology improves the solubility of hydrophobic drugs due to an increased surface area to volume ratio and improved dissolution rates (i.e., dissolution velocity) associated with nanosizing.^8^ The drug crystals are singularly well‐suited for the rehabilitation of previously unsuccessful Biopharmaceutics Classification System (BCS) Class II and IV drugs (low solubility drugs).^9^ The BCS classification system is an experimental model that measures permeability and solubility under prescribed conditions. The system divides the drugs into four classes. While Class I drugs have high solubility and high permeability, Class II molecules have low solubility and high permeability, Class III identifies with high solubility and low permeability, and drugs in Class IV have low solubility and low permeability.^4^

Nanocrystal drug formulations have also been shown to be stable in suspensions and are often referred to as nanocrystal colloidal dispersions (NCD's). The dispersions provide a platform for easy scale‐up and manufacturing of highly stable and marketable products. Their synthesis and scale‐up considerations have been described at length elsewhere.^10^, ^11^ Commonly used synthesis techniques include the use of microfluidic based platforms or the milling method, which, among others, is both flexible and tunable.^7^, ^12^, ^13^, ^14^, ^15^, ^16^, ^17^ Taken together, the nanocrystal drug technology has been studied extensively and is well positioned for further exploration in the field of drug delivery.

Several hydrophobic drugs have been salvaged via the nanocrystal formulation method. The drugs were successfully developed, and approved by the FDA to treat a variety of indications ranging from dental disorders to cancer in the clinic.^14^, ^18^, ^19^, ^20^, ^21^, ^22^, ^23^, ^24^, ^25^, ^26^, ^27^, ^28^ Depending on the disease, the approved formulations can be administered via different routes including oral, dermal, and parenteral. This highlights the versatility of a nanocrystal drug platform. Pharmacokinetic, biodistribution, and bioavailability data for organs involved in delivery routes tested using nanocrystal technology have been addressed at length previously.^10^, ^13^, ^18^, ^24^, ^25^, ^29^, ^30^, ^31^, ^32^, ^33^, ^34^ Specifically, the reviews of Lu et al. 2016 and 2017 delve into the biodistribution pattern of nanocrystal drugs in the blood, heart, liver, spleen, lung, kidney, tumor, and thymus (i.e., the organs involved in clearance/circulation and host immune responses).^24^, ^35^

Several articles have been published, discussing the techniques used to synthesize nanocrystal drugs; the type of stabilizers or surfactants involved; and the methods adopted for physicochemical and biological characterization.^10^, ^19^, ^36^, ^37^ However, a wide translational gap exists between this highly promising platform and its clinical approval. In this review, we discuss the nanocrystal drug technology and its development from a translational perspective. We speak to the paucity of FDA approved products despite the platform's obvious strengths. We discuss the challenges involved in their successful translation to the clinic.

## 2 PREPARATION AND CHARACTERIZATION OF NANOCRYSTALS

Properties such as crystallinity, size, shape, surface charge, and the type of stabilizers or polymer coatings used during formulation influence the therapeutic outcome of nanocrystal drug products. Other physicochemical properties currently under investigation for their influence on preclinical (i.e., in vivo*/*in vitro) performance assays include stiffness and surface texture.

### 2.1 Nanocrystal drug dissolution: Concept and theory

Although often overlooked, crystallinity is a foundational parameter for drug nanocrystals. It can provide insights into the structure of the final formulation. Assessing crystallinity is critical in verifying the successful integration of stabilizers, surface polymers (chemically conjugated or physically adsorbed), and targeting ligands. Further, nanocrystals with an amorphous crystalline substructure have an increased dissolution rate and are better suited for delivering multiple hydrophobic drugs.^21^, ^38^, ^39^ This is better explained using the “spring and parachute” concept adapted to describe dissolution rates of amorphous, crystalline, or co‐crystalline drugs.

**Figure 2:**
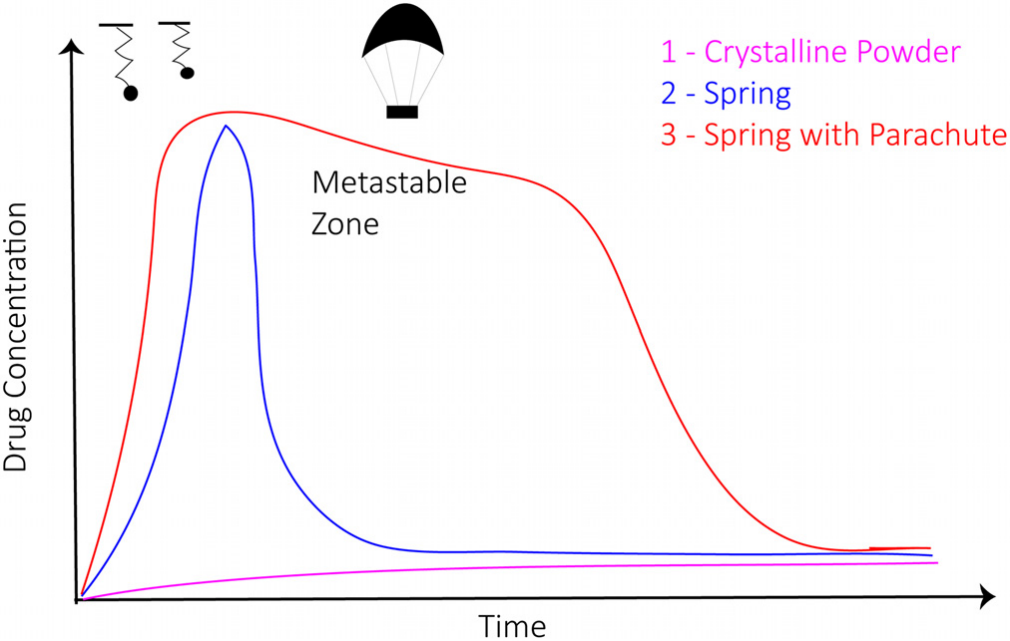
Schematic depicting spring and parachute model for nanocrystal dissolution as a function of time and drug concentration (Adapted from N. Babu and A. Nangia, 2011, Cryst Growth Des, 11, 2662–2679)

Co‐crystalline drugs are often composed of multiple components, including a hydrophobic drug and a stabilizer. Stabilizers are supplementary molecules which when added during formulation, can control the nanocrystal size, agglomeration, and its overall biodistribution in vivo.^8^, ^14^ Stabilizers are generally 50–500 fold more soluble in water than the drug in its free powder form. When exposed to an aqueous environment, the stabilizer first begins to leach into solution and leaves behind the drug particles. Subsequently, the loosely self‐aggregated drug particles form supramolecular aggregates. The co‐crystal at this point is in an amorphous crystalline state resulting in a high peak concentration (Figure 2). The highly soluble amorphous co‐crystal slowly transforms to a stable species following Ostwald's law that promotes dissolution into a free form of the drug (Curve 1). The time required for the drug to move through the high‐energy aggregate stage to a low‐energy solubilized state exhibit a “parachute effect.” Here, a high dissolution rate is achieved over an extended period of time. In absence of the stabilizer, the drug precipitates rapidly to a stable polymorph via the so‐called “spring effect.” This results in a modest improvement in solubility which is short‐lived and not sustained for long‐term dose regimens. The stable crystalline drug is almost insoluble as shown by Curve 1.

### 2.2 Physical analytical methods to characterize nanocrystal drug products

Stabilizers that are widely used in nanocrystal drug formulations are presented in Table 1. These are amphiphilic molecules that increase the nanocrystal's surface wettability, and when used at optimal concentrations, do not interfere with the crystal growth. Its successful integration into the final formulation can be confirmed simultaneously with the crystal's substructure using X‐Ray spectroscopy methods such as Small‐angle X‐ray scattering, X‐ray diffraction, and X‐ray photoelectron spectroscopy.

**Table 1:** Examples of drug/stabilizer combinations in nanocrystal formulations (compiled from Refs. 40, 41, 42, 43, 44, 45, 46, 47, 48, 49, 50)

Nanocrystal Core molecule	Stabilizer	Process
Glibizide	Sodium lauryl sulfate, Polyvinyl pyrrolidone K30, Pluronics F68 and F127, Tween 80, hydroxypropyl methylcellulose	Milling, Antisolvent precipitation
MTKi‐327	Pluronic F108, Lipid S75,	Milling
Beclomethasone diproprionate	Hydrophobin	Antisolvent precipitation
Naproxen	Vitamin E tocopherol polyethylene glycol succinate, Pluronic F127, sodium lauryl sulfate, di(2‐ethylhexyl) sulfosuccinate,	Milling
Paclitaxel	Hydroxylpropyl methylcellulose, polyvinylpyrrolidone, polyethylene glycol 400, Pluronics F127 and F68, sodium lauryl sulfate, Tween 20 and 80, transferrin, immunoglobulin G, human serum albumin	Antisolvent precipitation, sonication
Indomethacin	α‐, β‐, and γ‐ cyclodextrans, Pluronics F68, 17R4, and L64, Tetronics 908 and 1107	Emulsion solvent diffusion, milling
Budesonide	Lecithin, Pluronic F68	Milling
Curcumin	Polyvinyl alcohol, polyvinyl pyrrolidone, vitamin E tocopherol polyethylene glycol succinate, sodium lauryl sulfate, Carboxymethylcellulose sodium	High pressure homogenization
Nitrendipine	Polyvinyl alcohol	Antisolvent precipitation, ultrasonication
Brinzolamide	Tween 80, Pluronics F68 and F127, Hydroxypropyl methylcellulose	Milling
Fenofibrate	Hydroxypropyl methylcellulose, Soluplus	Milling
Nimodipine	Pluronic F127, Hydroxypropylmethylcellulose	Microprecipitation, high pressure homogenization
Loviridine, cinnarizine, griseofulvin, mebendazole, phenylbutazone, phenytoin	Polyvinyl pyrrolidone, polyvinyl alcohol‐polyethylene glycol, Pluronic F68, tocopherol polyethylene glycol succinate, hydroxypropyl methylcellulose, hydroxyethyl cellulose, hydroxypropyl cellulose, methylcellulose, carboxymethylcellulose sodium, polyvinyl alcohol, sodium alginate, tween 80	Milling
Cellulose	Tetradecyltrimethylammonium bromide, dodecyldimethylammonium bromide, cetyltrimethylammonium bromide, Hexadecyltrimethylammonium	Miniemulsion polymerization, Pickering emulsions
PH‐797804 MAPK inhibitor nanocrystal‐polymer particle	Polylactic acid	Miniemulsion polymerization
Gold	Cetyltrimethylammonium bromide, benzyldimethylhexadecylammonium chloride, hexamethylenetetramine, polyvinyl pyrrolidone, polyethylene glycol, β‐cyclodextrins	Self‐assembly, antisolvent precipitation
Cobalt	Trioctylphosphine oxide and oleic acid	Self‐assembly, antisolvent precipitation
Cyclosporin A	Imwitor®900, Tagat®S, sodium cholate	High pressure homogenization
Dexamethasone, ibuprofen, tacrolimus	Poloxamer 407, Poloxamer 188, vitamin E‐TPGS, lecithin, Plantacare 2000 UP	Wet bead milling
IONP	Poly(ethylene glycol)–poly(vinylphosphonic acid) block copolymers (PEG‐*b*‐PVPA)	Antisolvent precipitation

Nanocrystal drug sample preparation for X‐Ray spectroscopy analysis often involves freeze‐drying. The effects of freeze‐drying on the agglomeration of nanocrystals and its subsequent re‐dispersibility have been studied and reported previously.^51^ Liquid nanocrystal dispersions designed for dosing in their solid form must be freeze‐dried *above* the critical freezing rates. Using lower rates would increase particle aggregation and affect formulation redispersability.^52^ Critical freezing rates may be determined by treating re‐dispersibility as a result of competition between the freezing speed and the particle collision frequency. With increase in drug concentrations, the average inter‐particle distance decreases, thereby increasing the frequency of collisions. This results in an overall increase in the critical freezing rate.^52^ Thus, understanding the role of freeze‐drying in determining nanocrystal drug stability is critical during translation.

Methods commonly used to confirm the incorporation of stabilizer, polymer coating, or targeting ligands during co‐crystallization include Fourier Transform Infrared spectroscopy, Raman spectroscopy, and Nuclear Magnetic Resonance spectroscopy. The aforementioned techniques are among the most preferred methods for characterizing nanocrystal compositions. In a review by Luykx et al.,^53^ analytical techniques that may be used to characterize size, shape, charge, and the composition of NP drugs have been described. Some of these methods are deemed as facile and are not as widely used in nanocrystal drug development. These include field flow fractionation for size, desorption electrospray ionization‐mass spectrometry, and ion‐mobility spectrometry‐mass spectrometry for mass and composition, photon correlation spectroscopy for size and distribution, and analytical ultracentrifugation for size, shape, and structural analysis.

Previous studies have probed the effects of NP size, shape, surface morphology, and charge on therapeutic outcome. It has been shown that these parameters influence phagocytosis, immune responses, endothelial targeting, adhesion under flow, transport mechanisms, and intracellular delivery.^54^, ^55^, ^56^, ^57^, ^58^, ^59^, ^60^, ^61^, ^62^, ^63^ As an example, Mitragotri and co‐workers described the differences in internalization rate and the pathways of NPs differing in shape, size, and aspect ratio in mouse peritoneal macrophages.^58^ Large particles (>100 nm) are usually internalized via non‐specific pathways such as phagocytosis and macropinocytosis. However, nanocrystal drug surfaces can be modified to minimize non‐specific uptake and facilitate entry via specific pathways such as receptor‐mediated endocytosis. This can be achieved by coating the surface with polymers or surfactants such as PEG, PEG derivatives, polydopamine, and Pluronic F127 or with antibody coatings. Chung et al. showed that coating iron oxide NPs with positively charged multi‐arm PEG derivatives could reduce mass aggregates and used to uniquely label mesenchymal stem cells.^64^ Jiang et al. showed that a positive charge on iron oxide NPs coated with lipids can deliver nucleic acid payloads to cells.^65^ Sonvico et al. showed that dextran coatings on maghemite NPs can achieve comparable disaggregation properties to PEG coatings and can be used to conjugate targeting moieties.^4^ It has also been shown that surface modifications can significantly affect the dissolution kinetics of pure drug NPs.^66^, ^67^, ^68^, ^69^ Stiffness and surface texture are also known impact the in vivo performance of NPs.^62^ Eliaz et al. and Lorenzetti et al. showed that nanocrystals developed for bone grafting and other bone related therapeutic applications (i.e., joint reconstruction) are particularly sensitive to stiffness (i.e., rigidity), surface texture (i.e., roughness), and hardness. The properties are influenced by bone‐forming cells and are components crucial to the successful integration or rejection of these materials.^70^, ^71^ These findings highlight the importance of translational aspects of physical forces and properties that impact the biological performance and its clinical relevance. Table 2 summarizes the different sizes and geometries which have had success in preclinical research models. The table highlights the diversity of size domains, morphologies, and type of nanocrystal (i.e., organic/inorganic).

**Table 2:** Nanocrystal variants with a varied range of properties including composition, size, and geometry

Type of nanocrystal	Size	Geometry	References
Iron oxide	11–16 nm	Spheres	72
Copper nanocrystals	2–10 nm	Spheres Cylinders Tetrahedra Cubes	73
Gold nanocrystals	1–4 nm	Spheres	74
Cellulose nanocrystals	141–1,073 nm (length) 12–28 nm (width) 1.12–1.45 nm (pitch)	Rods Ribbons	75
Hydroxyapatite	10–12 nm (width)	Plate‐like crystals	76
Hydroxyapatite	200 × 40 nm	Plate‐like crystals	77
Camptothecin	200–700 nm	Rods Needles	78
Lutein nanocrystal	429–560 nm 1–3 μm	Spheres in suspension Spherical capsules	79
Chitosan/LaF_3_:Eu^3+^	13–18 nm	Spheres	80
DNA‐Au nanocrystal hybrids	<20 nm	Spheres	81

**Figure 3:**
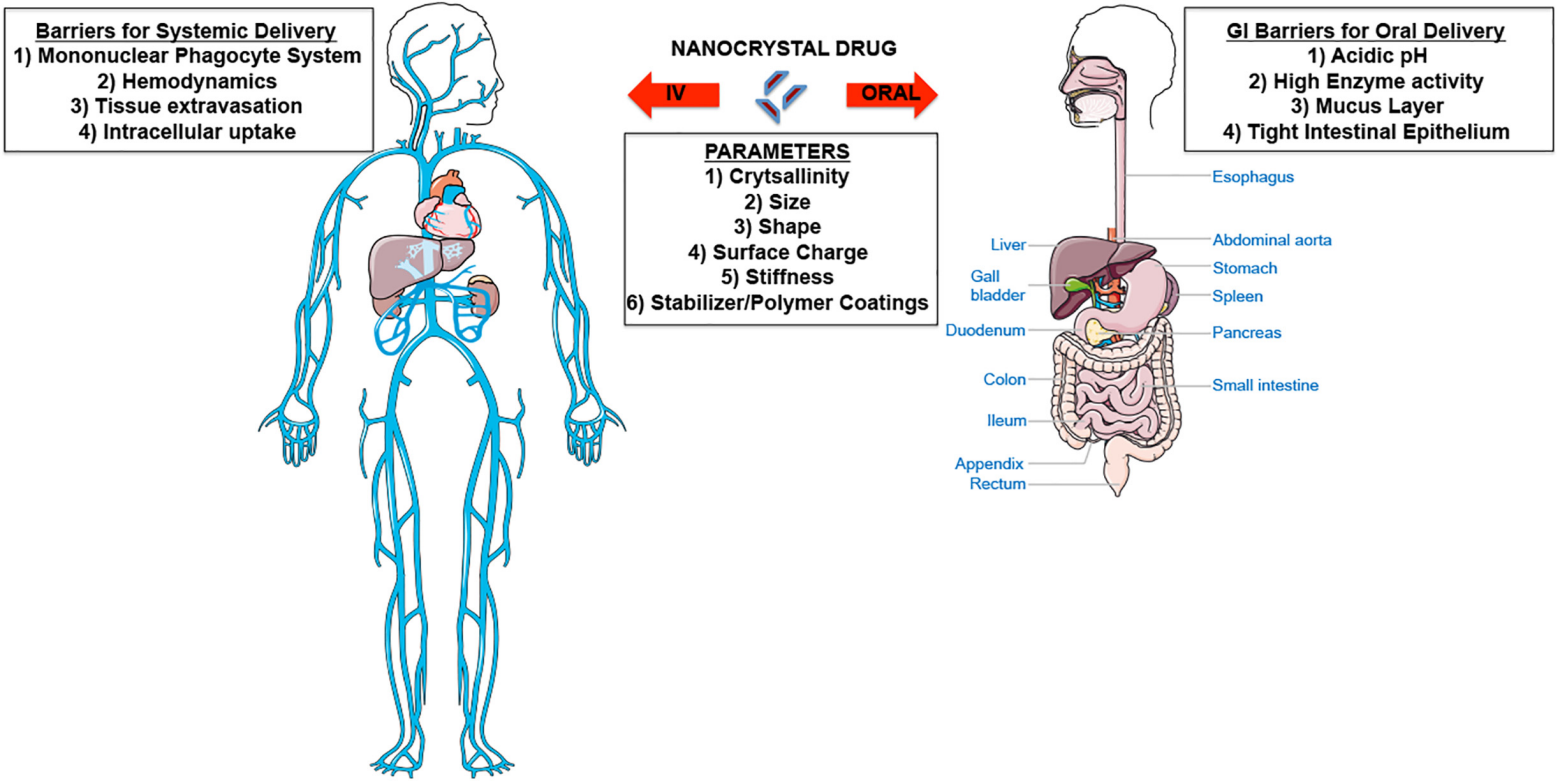
A schematic depicting the in vivo barriers and properties that influence in vivo biodistribution and site‐specific delivery of nanocrystal drug products administered orally or intravenously (IV)

The influence of surface charge on the in vivo fate of NPs has been extensively researched and reported. For instance, positively charged iron oxide nanocrystals have been found to induce cytotoxic effects in vitro in a charge‐dependent manner. This is believed to result from increased endocytosis due to the strong binding between the positively charged surface on the crystal to the negatively charged glycolipid membrane. The internalized positively charged nanocrystals further interact with the negatively charged organelles and DNA.^82^ A similar observation was also noted for paclitaxel nanocrystals where a positive charge led to higher cell uptake and cytotoxicity compared to negatively charged particles.^66^ On the contrary, negatively charged nanocrystals exhibited significant uptake via clathrin‐ and caveolae‐mediated uptake mechanisms.^83^ Additionally, excessive positive and negative surface charges on NPs have been shown to induce higher rates of opsonization and capture by the immune cells in vivo.^83^ A vastly diverse array of nanocrystalline material fabricated with various surface charges for drug delivery purposes has been explored and reported in the literature (Table 3). Finally, a majority of nanocrystal drug products approved for use in the clinic and or in clinical trials are delivered via oral or intravenous administration. Figure 3 depicts the challenges faced by these products to overcome the various biological barriers in vivo and properties that influence its biodistribution and site‐specific delivery.

**Table 3:** Nanocrystals with varied surface charges

Type of nanocrystal	Preclinical model (in vitro)	Surface charges	References
Magnetite	HCT116, NIH3T3	Reported as neutral	84
SPIONS^a^	HUVEC MCF7	+9.4 mV, −8.3 mV	85
Cellulose‐FITC Cellulose‐RBITC	HEK 293, *Sf9*	+3.9 mV, −46.4 mV, −48.7 mV +9.0 mV, 8.7 mV, 8.6 mV	86
Cellulose	KU‐7	0 mV to −55 mV	87
Cellulose	MDCK, HeLa, Caco‐2, J774	−40 mV to 100 mV	88
Camptothecin	Eahy926 4T1	−4.67 mV, −9.66 mV, −30.4 mV	89
Paclitaxel	A549	+19.3 mV, −2.4 mV, −22.7 mV	90
Paclitaxel	MCF‐7, HaCaT	−2.73 mV, −15.9 mV, −16.6 mV	91
Hydroxyapatite	MC3T3‐E1	−12.5 mV, −23.3 mV	92
Hydroxyapatite	MC3T3‐E1	+48.6 mV, −11.4 mV, −28.3 mV	93
NC‐quantum dots	Vero	+40.52 mV, +36.2 mV, −48.12 mV −55.15 mV, −58.75 mV	94
Lanthanide Doped	HT29, OVCAR3, Wistar rats	Reported as positive	95
CdSe quantum Dot NC–MUA ligand	NHBE cells	−21 mV, −53.5 mV, −71.8 mV	96
CdSe quantum Dot NC–MPA ligand	NHBE cells	−29.4 mV, −39 mV, −56 mV	96
CdSe quantum Dot NC–AUT ligand	NHBE cells	+86.8 mV, 73.7 mV, 60.6 mV	96
CdSe quantum Dot NC–CYST ligand	NHBE cells	+57.4 mV, 46.7 mV, 43.4 mV	96
Cerium oxide (nanoceria)	H9c2, HEK293, A549, MCF‐7	+30 mV, 0 mV, −45 mV	97

a SPIONS, superparamagnetic iron oxide nanoparticles. Surface charges listed left to right as positive, neutral, negative; respectively.

## 3 NANOCRYSTAL‐DRUG PRODUCTS

Nanocrystals, on account of their high‐drug loading efficiency, steady dissolution rates, enhanced structural stability, and extended circulation times, have been a topic of high research and development activity. Several products are already in the market, and a number of other formulations are undergoing clinical trials.

### 3.1 Fabrication techniques for nanocrystal‐drug products

Nanocrystals are typically produced using two approaches: (a) top‐down and (b) bottom‐up approaches. Top‐down approaches include methods such as media milling (pearl milling) and high‐pressure homogenization (HPH). Here the large‐sized particles are broken down to a smaller size. In media milling, the milling chamber is filled with milling pearls, the stabilizer, drug, and the dispersion media. The milling pearls are usually made of stainless steel, glass, zirconium oxide, or highly cross‐linked polystyrene resin. The drug‐crystals are nanosized by a combination of collision with the milling pearls; the milling chamber; and high shear forces. During HPH, drug particles—raw or dispersed in aqueous media with surfactant—are transformed into micronized suspensions by a sonicator, homogenizer, mortar, and pestle or jet mill.^98^, ^99^, ^100^, ^101^, ^102^ The suspensions are then exposed to a series of collisions, strong cavitation, and high shear forces generated by passing through a narrow gap. This causes it to boil due to change in pressure differences. For a bottom‐up approach, the most common method is precipitation. Here, nanosuspensions are engineered from completely dissolved small molecules in their corresponding antisolvent. It involves two steps—nucleation and crystal growth. Other methods include the use of an acid–base neutralization step. Here nanosuspensions are prepared from a drug dissolved in an acid‐organic solvent mix and then added gradually to a base until the solution is neutralized.^103^ Nanosuspensions have also been prepared using techniques such as microfluidic nanoprecipitation process, spray drying, electrospraying, and aerosol flow reactor.^104^, ^105^, ^106^, ^107^, ^108^ The pros and cons of both top‐down and bottom‐up approaches are summarized in Table 4.

**Table 4:** Top‐down versus bottom‐up approaches for nanocrystal‐drug products

	Technique	Merits	Limitations
Top‐down approaches	Media milling (MM)	1. Works for drugs that are insoluble in both aqueous and non‐aqueous solvent. 2. no organic solvents are used 3. ease of scale‐up 4. minimal batch to batch variation 5. narrow size distribution of particles 6. high drug loading efficiency	1. Costly manufacturing process 2. high energy requirements with long durations for milling 3. could destabilize the drugs due to high shear forces and temperature 4. risks for contamination from the dispersion media 5. unwanted drug loss
	High‐pressure homogenization (HPH)	‐ Same as MM ‐	1. Particles need to be micronized and form suspensions 2. risk of contamination including machine debris 3. high energy requirements
Bottom‐up approach	Precipitation	1. Simple and less expensive 2. minimal energy requirements 3. ease of scale‐up 4. possible non‐stop production	1. Extensive optimization required selecting solvent/antisolvent 2. possible growth of particles with time 3. inadequate purification process or removal of toxic solvents

### 3.2 Nanocrystal‐drug products in the market

Since 1995, the FDA has approved ~50 nanodrugs, mostly based on liposomes, polymers, and nanocrystals for various indications.^109^, ^110^ Nanocrystallization is an effective way to formulate and develop poorly soluble drugs. The commercial value of this technology is further enhanced by the relatively short span of time to clinical approval. While liposomes took almost 25 years to commercialize, the relative development time for Emend® was just 10 years. Emend's first patent application was filed in 1990 and the product was launched in 2000.^29^ Thus, compared to other nanosized platforms, a large number of nanocrystal drug products have been developed and launched successfully within a limited span.^29^

Rapamune®, a poorly soluble immunosuppressant Sirolimus (SRL), was the first marketed nanocrystal drug product, introduced by Wyeth Pharmaceuticals (Madison, NJ) in the year 2000. Rapamune was formulated using the pearl mill technology method, and its oral bioavailability was found to be 21% higher than SRL in its conventional oral solution form. This was followed by the launch of Emend (Aprepitant), in 2003 by Merck (Winehouse Station, NJ). Emend was formulated from Aprepitant—a poorly water soluble anti‐emetic medication, which can only be absorbed in the upper gastrointestinal tract and has a narrow absorption window. Nanoionization of Aprepitant via the pearl mill technology increased its oral bioavailability by making it more soluble in water. Tricor®, launched by Abbott Laboratories in 2003, was formulated from fenofibrate—a lipophilic medication for Hypercholestremia—using the pearl mill technology method. Formulating fenofibrate into nanocrystals increased its adhesiveness to the gut wall and improved its oral bioavailability by 9% independent of fed or fasted state. This made way for a simplified, flexible dosing regimen for patients. Another nanocrystal drug product, derived from fenofibrate is Triglide® which was launched by Skyepharma in 2005. The Triglide nanocrystals were produced using the HPH method and provided benefits similar to Tricor. Triglide achieved an improved bioavailability that was independent of the fed or fasted state with increased adhesiveness to the gut wall. Triglide is currently marketed by Sciele Pharma Inc. (Atlanta, GA). Another nanocrystal product is Megace ES® and was launched by Par Pharmaceutical Companies, Inc. (Spring Valley, NY) in 2005. Megace ES was formulated into nanocrystals from megestrol acetate—an appetite stimulant using the pearl mill method. This improved its dissolution rate and reduced the single dose volume by four times, thereby improving its oral bioavailability and patient compliance when compared to the highly viscous megestrol acetate oral suspension. Other approved nanocrystal drug products are listed in Table 5 and media milling is the most widely accepted method used to produce a majority of the marketed products.

**Table 5:** Nanocrystal drug products in the market^29^

Trade name	Company	Drug	Indication	Technology	Delivery route	Approval year
Rapamune	Wyeth	Rapamycin/sirolimus	Immunosuppressive	Coprecipitation	Oral	2000
Emend	Merck	Aprepitant	Anti‐emetic	Media milling	Oral	2003
Tricor	Abbott	Fenofibrate	Hypercholesterolemia	Media milling	Oral	2004
Triglide	Skye Pharma	Fenofibrate	Hypercholesterolemia	High pressure homogenization	Oral	2005
Megace®ES	Par Pharma	Megestrol acetate	Appetite stimulant	Media milling	Oral	2005
Invega Sustenna®	Johnson & Johnson	Paliperidone palmitate	Antidepressant	High pressure homogenization	Parenteral	2009
Cesamet®	Lilly	Nabilone	Anti‐emetic	Coprecipitation	Oral	2009
Avinza®	King Pharma	Morphine sulfate	Anti‐chronic pain	Media milling	Oral	2002
Naprelan®	Wyeth	Naproxen sodium	Anti‐inflammation	Media milling	Oral	2006
Ritalin LA®	Novartis	Methylphenidate hydrochloride	Anti‐psychotic	Media milling	Oral	2002

**Table 6:** Nanocrystal drug products in clinical trials

Trade name	Company	Drug	Indication	Technology	Delivery route	Clinical status
Semapimod	Cytokine Phamasciences	Guanylhydrazone	TNF‐α Inhibitor	Self‐developed	Intravenous	II
Paxceed®	Angiotech	Paclitaxel	Anti‐inflammatory	Unknown	Intravenous	III
Theralux	Celmed	Themectacin	Autoimmune diseases and cancer	Media milling	Intravenous	II
Nucryst®	Nucryst Pharmaceuticals	Silver	Atopic dermatitis	Self‐developed	Topical	II
PanzemNCD	EntreMed	2‐methoxy estradiol	Ovarian cancer	Media milling	Oral	II

### 3.3 Nanocrystal drug‐products in clinical trials

As seen in Table 5, a majority of nanocrystal drug products are currently approved for oral ingestion and treating diseases other than cancer. The market for oral administration is enormous and, the path to commercialization is easier compared to injectables. Since the product is primarily composed of the drug and can be incorporated with GRAS approved stabilizers and excipients, the regulatory approval process for nanocrystal drug products is easier. Thus considering the feasibility for rapid development and commercialization, there are several nanocrystal drug products currently in clinical trials, as referred to in Table 6. Semapimod® nanocrystals from Cytokine Pharamsciences (CPSI) is a synthetic guanylhydrazone and was found to act as an immunomodulator, preventing the production of TNF‐α, a proinflammatory cytokine, involved in inflammation‐associated carcinogenesis during a Phase I study in cancer patients.^111^ CPSI also showed the drug to be effective in treating psoriasis and moderate‐to‐severe Crohn's disease during a preliminary clinical trial. Another nanocrystal drug currently in clinical trials is PAXCEED™ from Angiotech Pharmaceuticals, Inc..^112^ PAXCEED is formulated from paclitaxel and is a cremophor EL‐free systemic formulation. This could potentially reduce hypersensitivity in patients treated for cancer or chronic inflammation. Theralux™ from Celmed BioSciences Inc., (Saint‐Laurent, QC) is a photodynamic therapy‐based treatment system consisting of thymectacin, which is poorly soluble and has minimal bioavailability.^113^ It is currently being evaluated in autoimmune diseases, non‐Hodgkin's lymphoma, colon cancer and prevention of graft‐versus‐host disease. Nucryst Pharmaceuticals (Wakefield, MA) has developed a cream formulation based on a proprietary substance NPI 32101, which is primarily composed of silver nanocrystals.^114^, ^115^ The drug displayed promising anti‐inflammatory and antimicrobial properties.^116^ NPI 32101 is currently undergoing Phase II clinical trials for atopic dermatitis.^117^ Panzem® NCDs were formulated from 2‐Methoxyestradiol (2‐ME2)—a natural metabolite of estradiol (EntreMed, Inc.). 2‐ME2 showed promising antiproliferative and antiangiogenic properties during preclinical trials. Subsequently, Entremed moved ahead with Panzem to test its activity against ovarian cancer, and other solid carcinomas. However, it did not proceed beyond Phase II, and all clinical development of Panzem was suspended.^118^

### 3.4 Challenges and criteria in the clinical development of nanocrystal‐drug products

The past two decades witnessed major strides being made in the development of nanocrystal drug technology. A majority of the active pharmaceutical ingredient (API) used for these formulations have low water‐solubility. This, in turn, affects the drugs' systemic bioavailability post‐administration causing inconvenient dosing regimens for patients. Nanoionization of such APIs can result in low particle size, with increase in surface/volume ratio and rate of dissolution. Consequently, this could improve dose proportionality, linear pharmacokinetics, and bioavailability when compared to its original composition. Physicians could then predict the therapeutic response and customize dosing regimens for individual patients. However, issues pertaining to the quality, Chemistry, Manufacturing, and Controls, or bioequivalence (BE) of nanocrystal drug products still exist during scale‐up and development. This could delay the clinical advancement of promising nanocrystal drug products for a variety of indications.

One such issue is determining the most appropriate conditions for testing in vitro dissolution rates of newly formulated products. Nanocrystal drug dissolution usually occurs in two steps—(a) the drug molecules are released from the crystal surface into the surrounding dissolution media to create a saturated layer close to its surface; (b) the released molecules then diffuse through the solvent from a region of high concentration (i.e., the saturated layer) to a region of low concentration. Assessing the dissolution rate of a newly formulated nanocrystal drug product is vital and useful during its development and manufacturing process. It would meet the requisites for worldwide regulatory standards while establishing safety, efficacy, and quality. Depending on the delivery route, dissolution studies must be carried out in vivo or in conditions that simulate in vivo. For instance, if delivered orally, the drugs need to be released from the crystal, absorbed by the gastrointestinal (GI) tract and circulate in the blood to reach its site of action. The study design should take into account the harsh gastric conditions including the acidic pH (1–3), constant churning, the intestinal compartment's pH (5–7), and so forth.

The Noyes–Whitney's equation is often used to describe the drug dissolution rate at a specific time.^119^

$$\frac{\mathrm{Dx}}{\mathrm{dt}}=\frac{A.D}{\partial }.C_{s}-\left(\frac{X_{d}}{V}\right),$$

where *Dx/dt* is dissolution rate, *A* is surface area of the dissolving particle, *D* is the diffusion rate constant, *δ* is thickness of the stagnant layer surrounding the particle, *Cs* is saturation solubility of the drug, *X*_*d*_ is amount of drug dissolved at time *t*, and *V* is volume of the dissolution media.

Thus, if the dissolution media increases the drug solubility, it should also increase the crystal's dissolution rate. Thus, it is crucial to use conditions that closely mimic the physiological environment under in vitro conditions. Current dissolution techniques such as the paddle‐method combined with UV spectroscopy or HPLC do not mimic physiological conditions. A high degree of variability therefore exists between in vitro dissolution and in vivo bioavailability data. Use of microfluidic co‐culture devices that take physiological considerations into account could facilitate a smoother translation into the clinic.^69^, ^120^, ^121^ It would be useful to assess the quality of the product and predict its in vivo performance. Consequently, this will reduce the number of BE studies performed in humans during the clinical development, scale‐up, and post‐approval changes.

Other challenges that exist during product development relate to: (a) control of drug substance, (b) control of drug product, (c) manufacturing process, and (d) stability of drug substance/drug product. Depending on the purity, drug substance, is referred to as the API without excipients and achieves the end therapeutic effect. The effect depends on physical attributes such as size and crystallinity. This could affect the manufacturing process and quality of the final product. Since particle size and distribution depend on the dispersion media and stabilizers used during formulation, values are reported in terms of particle size distributions (*D* values). The *D* values represent the midpoint values and range during submissions. When suitable, specifications are expressed as intensity‐weighted harmonic mean (*Z*‐average) and polydispersity index with histograms. Another concern would be identifying the ideal technique to validate particle size. To ensure reliability, at least two analytical methods that support each other should be used to determine particle size and distribution. Dependence on pH may also be taken into account since nanocrystal size and stability are often affected by pH of the dispersion media. In terms of crystallinity, change in structure to amorphous or polymorphic variants could affect its dissolution, stability, and bioavailability. It is therefore important to ensure that the change in crystalline structure is monitored and controlled during manufacture and shelf life of the final product.^122^, ^123^, ^124^, ^125^, ^126^, ^127^, ^128^, ^129^

Drug product is referred to as the prototype or the marketed dosage form of the drug substance formulated with excipients. Its control refers to factors that affect the quality and in vivo performance of the final product. This is often affected by changes in viscosity, dissolution rate, specific gravity, content uniformity, and redispersability. The factors are also influenced by the presence of impurities formed during manufacturing. Hence, due consideration must be given for assays that test the purity and continuously monitor degradation products formed during manufacture or shelf life of the final product.

Factors critical during the product's manufacturing process include determining the most appropriate tests or controls to monitor particle size distribution, agglomeration, and presence of contaminants at various steps during the process. It is therefore important to continuously track impurities generated during the process.^29^, ^130^ In case of a top‐down approach as in wet milling, impurities depend on the milling media used, the milling material that comes in contact with the drug, the milling mechanism and the number of milling cycles used for the process. Further, other factors such as product and chiller temperature as a function of time, the drive motor speed, shape, aspect ratio, viscosity of product dispersion, and so forth may affect the process and particle size distribution.^27^, ^29^, ^130^ In case of a bottom‐up approach, care should be taken to ensure that (a) a uniform dispersion of the drug is maintained and agglomeration is prevented while adding the drug slowly in to the melt, (b) an optimal viscosity is maintained for the molten material, (c) consistency is sustained during sampling and the solidification/cooling procedure and finally, (d) solvent residues and other impurities in the drug substance/product are tracked and isolated on a constant basis.^131^ Deviations in any of the above steps could significantly impact product quality, and affect its in vivo performance.

Since crystallinity of the final product could significantly impact its quality and in vivo performance, it is important to ensure that structural stability is retained post‐manufacturing and throughout its shelf life. As stated previously, techniques such as X‐ray powder diffraction, differential scanning calorimetry, or spectroscopic methods, and so forth can be used to study and compare the structure of the initial molecule with the final product and at the end of its shelf life. Additional studies may be designed concerning short or long‐term stability, or its dosage form since particles tend to aggregate due to sedimentation, redispersion, or caking, and so forth.

## 4 NANOCRYSTAL DRUGS: A CARRIER‐FREE THERAPEUTIC PLATFORM FOR CANCER AND OTHER DISEASES?

By the year 2021, nanocrystal drug products are estimated to account for 60% of the total NP‐based drug delivery market.^132^ This is valued to be at ~$82 billion. While nanocrystal technology is attractive due to its ease of formulation, uniform composition, and attractive pharmacoeconomic values, it also has the potential to overcome some of the biggest challenges for drug development. Poor solubility could result in abysmal bioavailability, thereby affecting optimal delivery of the drug. By formulating poorly soluble drugs into nanocrystals, the resulting increase in surface/volume ratio, saturation solubility, and the rate of dissolution can ensure an enhanced bioavailability of most insoluble drugs irrespective of its route of administration. Clinical efficacy of nanocrystal drugs depend on several factors including the size, morphology, surface charge, amount of drug loaded, the type of excipient used, the degree of redispersability, and site‐specific targeting. Also, multimodal theranostic nanocrystal drug products would be vital to assess and monitor in vivo bioavailability. A majority of nanocrystal drug products are currently approved for oral ingestion and for treating diseases other than cancer. The manufacturing process for such oral products is consistent and form crystals at sizes well above the 100–200 nm range. Since nanocrystals are not expected to undergo rapid dissolution in the blood due to a minimum volume of distribution; they can be injected intravenously. However, crystals with dimensions >100–200 nm could promote macrophage‐mediated phagocytosis, rapid blood clearance, and minimal efficacy compared to the drugs' conventional form. Efforts must, therefore, be directed to develop crystal products at sizes well below the 100 nm range with surface modifications to avoid renal clearance and sequestration by the mononuclear phagocytic system. Besides, it is vital to understand their intracellular and intratumoral fate. Overall, nanocrystals have the potential to open up new frontiers in the field of therapeutics. The ability to reformulate off‐patent drugs into novel nanocrystal drug products for clinical use offers a clear competitive edge for companies in the market.
